# Microbial contamination and microbiome composition of fresh edible mushrooms: a critical review

**DOI:** 10.3389/fmicb.2026.1757755

**Published:** 2026-04-13

**Authors:** Aishiki Banerjee, Nitu Gupta, Apurba Koley, Muthusamy Senthil Kumar, Saurav Saha, Srinivasan Balachandran

**Affiliations:** 1 Biomarker Discovery Laboratory, The University of Kansas Medical Center, Kansas City, KS, United States; 2 Department of Environmental Science, Tezpur University, Tezpur, India; 3 Bioenergy Laboratory, Department of Environmental Studies, Siksha-Bhavana (Institute of Science), Santiniketan, India; 4 Department of Biotechnology, St. Xavier's College, Burdwan, India; 5 Department of Internal Medicine, The University of Kansas Medical Center, Kansas City, KS, United States

**Keywords:** edible mushrooms, food safety, microbial contamination, mushroom-microbiome, post-harvest spoilage

## Abstract

Fresh edible mushrooms have gained popularity as valuable dietary components, with global consumption steadily increasing due to their high nutritional and functional benefits. However, their constitutional characteristics make them especially vulnerable to microbial spoilage, potentially harming the fruiting bodies during cultivation and creating major challenges in harvesting, handling, and storage after harvest. This review highlights the types, sources, and impacts of microbial contamination in fresh edible mushrooms, with a focus on spoilage organisms. It explores the emerging field of mushroom microbiome research, highlighting the composition, diversity, and functional roles of microbial communities associated with 4 edible mushroom species (*Agaricus* sp., *Pleurotus* sp., *Lentinula* sp., and *Flammulina* sp). Studies employing high-throughput sequencing technologies to explore the microbial associations of edible mushrooms are discussed, providing deeper insights into these complex microbial ecosystems and their impacts on mushroom quality, shelf life, and safety. Bibliometric studies using VOSviewer over a 10-year period have uncovered global research trends, emerging focus areas, and identified gaps in the field. This review also discusses post-harvest control strategies and microbiome-targeted interventions to enhance microbial safety and extend shelf life. Edible mushrooms also contribute to the circular bioeconomy by converting agricultural residues into nutritious food. However, microbial contamination can compromise product quality and safety within this sustainable production system. Persistent knowledge gaps in understanding microbial dynamics and mushroom–microbiota interactions must be addressed to develop innovative, sustainable approaches to mushroom preservation and food safety management.

## 1 Introduction

Fresh edible mushrooms have been valued as nutritious foods for thousands of years, supporting human health and immune function. Among all known fresh edible mushroom species, roughly 25 are extensively cultivated (Riaz et al., 2022). Edible mushrooms are fleshy fruiting bodies that grow on soil or other organic substrates, and primarily belong to the fungal phyla Basidiomycota and Ascomycota (Bhagarathi et al., 2023). They are highly valued globally for their functional food properties, palatability, nutritional content, and medicinal potential (Assemie and Abaya, 2022; Chugh et al., 2022). Mushrooms are considered nutrient-dense foods, with protein accounting for 19–35% of the dry weight and carbohydrate comprising 50–65% of the dry mass. They are also rich in dietary fiber, essential amino acids, minerals, and bioactive compounds with immunomodulatory and antioxidant properties (Araújo-Rodrigues et al., 2024; Singh et al., 2025). The diverse bioactive properties of edible mushrooms are discussed in Table 1. The nutritional and bioactive characteristics of edible mushrooms have significantly strengthened their commercial relevance, driving the mushroom industry to intensify large-scale cultivation, invest in advanced production technologies, and expand its presence globally.

**Table 1:** Antimicrobial, antioxidant properties, and bioactive compounds of edible mushrooms.

Mushroom species/Genera	Bioactive compounds	Property type	Specific activity	References
General Edible Fungi	Bioactive compounds	Immunomodulatory/Antioxidant	Possess immunomodulatory and antioxidant properties.	Riaz et al. (2022) and Kamal et al. (2022)
General Edible Fungi	Various biomolecules	Pharmacological	Approximately 130 pharmacological properties have been identified, including immunostimulant, hepatoprotective, antioxidant, hypoglycemic, and anticarcinogenic activities.	Romero et al. (2025)
General Edible Fungi	High molecular weight compounds	Immunomodulatory	Include *β*-(1,3)-(1,6)-D-glucans, proteins, proteoglycans, and polysaccharide–protein complexes.	Romero et al. (2025)
General Edible Fungi	Low molecular weight compounds	Bioactive	Include terpenes, sterols, and ergosterol.	Romero et al. (2025)
*Pleurotus* spp.	Immunostimulants	Immunomodulatory	Capable of producing substances that stimulate the host’s immune system.	Romero et al. (2025)
*Pleurotus* spp. (Oyster Mushroom)	Lovastatin (Statins)	Health/Cardiovascular	Best known medically for their cardiovascular and cholesterol-controlling benefits.	Gupta et al. (2017)
General Edible Fungi	Bioactive compounds	Health/Medicinal	Medicinal values include wound-healing, immunity-enhancement, and tumor-retarding effects.	Rathore et al. (2017)
*Pleurotus ostreatus*	Antioxidant compounds	Antioxidant Activity	Showed high antioxidant activity, with an IC50 value of 100 μg/mL in the DPPH scavenging assay.	Chowdhury et al. (2015)
*Pleurotus ostreatus*	Unknown (methanolic extracts)	Antimicrobial Activity	Exhibits broad-spectrum antibacterial and antifungal activity; minimal inhibitory concentrations (MICs) were high for *Pseudomonas aeruginosa*.	Chowdhury et al. (2015)
*Lentinula edodes* (Shiitake)	Unknown (Methanolic extracts)	Antimicrobial Activity	Demonstrated the best antimicrobial activity among tested species, showing strong in-vitro antibacterial activity, for example, 17 ± 0.2 mm zone of inhibition against *Bacillus subtilis*.	Chowdhury et al. (2015)
*Lentinula edodes*	Phenols and flavonoids	Bioactive Compounds	Contained the highest levels of total phenols (10.66 ± 0.52 mg/mL) and flavonoids (4.76 ± 0.11 mg/mL) among tested species.	Chowdhury et al. (2015)
*Hypsizigus tessulatus*	Phenols and flavonoids	Bioactive Compounds	Contained total phenols (5.65 ± 0.05 mg/mL) and flavonoids (2.50 ± 0.008 mg/mL).	Chowdhury et al. (2015)
*Hypsizigus tessulatus*	Antioxidant compounds	Antioxidant Activity	Showed moderate DPPH scavenging activity, with an IC50 value of 105. ±1.23 μg/mL	Chowdhury et al. (2015)
*Volvariella volvacea, Auricularia, Flammulina, Tremella, Grifola*	Bioactive compounds	Health/Medicinal	Have varying degrees of immune system boosting, lipid lowering, anti-tumour, microbial, and viral properties.	Bhambri et al. (2022)
*Pleurotus* sp., *Auricularia* sp.	Esterases, oxygenases, oxidases/dehydrogenases	Biodegradation/Enzymatic	Enzymes in spent mushroom compost (SMC) are involved in organic pollutant biodegradation (e.g., phthalate removal), indicating potent metabolic activity.	Chang et al. (2021)

According to FAO (2023) reports, mushroom production has increased more than fivefold since 2000, reaching 44 million tons (Ludemann, 2024). The rising global demand for fresh edible mushrooms has driven increased production to meet retail market needs. In terms of country-specific production, China dominates with 70–75% of global output, followed by Japan and Poland (Bijla, 2023; Thakur, 2020). Six mushroom types primarily dominate global production and the market: shiitake mushroom (26%), oyster mushroom (21%), black ear mushroom (21%), button (11%), *Flammulina* (7%), paddy straw mushroom (1%), and other mushrooms (13%) (Bijla, 2023; Kamal et al., 2022). Nonetheless, the white button mushroom remains the most widely produced edible fungus worldwide, owing to its high nutritional value and rich content of bioactive compounds (Usman et al., 2021). However, demand for Portobello mushrooms (*Agaricus bisporus* var. *Portobello*), also known as brown button mushrooms, has increased significantly. In a context where health and sustainability are key consumer priorities, mushrooms offer notable functional benefits, including immune support, antioxidant properties, and improved gut health (Bell et al., 2022). As research into the underrecognized potential of mushroom-based diets expands, these dietary choices are gaining momentum among individuals seeking healthier, more natural, nutrient-dense options (Rathore et al., 2017). However, the growing commercial relevance of these mushrooms underscores the need to better understand the intrinsic physiological and microbial processes that drive their rapid postharvest deterioration.

Mushrooms undergo rapid water loss after harvest, leading to loss of turgor and textural softening, reduced freshness, and microbial spoilage (Mahajan et al., 2008; Huo et al., 2023). The edible fruiting bodies of mushrooms, characterized by high moisture content (water activity > 0.98), neutral pH, soft tissues, and nutrient-rich composition, provide an ideal environment for microbial proliferation, posing a challenge for postharvest maintenance. Species of *Pseudomonas* commonly thrive under humid conditions, colonize mushroom surfaces, and produce enzymes that degrade mushroom cell walls (Venturini et al., 2011). As a result, mushrooms develop slime, discoloration, unpleasant odors, and soft spots. Mushroom quality depends on various factors, including whiteness, texture, and microbial counts (Castellanos-Reyes et al., 2021). High bacterial populations in fresh edible mushrooms are a key factor reducing quality by causing brown blotches, and the rate of postharvest deterioration is directly related to the initial microbial load (Beelman et al., 1989). Generally, fresh edible mushrooms have a short shelf life, typically 3–10 days, depending on the species, storage conditions, and packaging (Feng et al., 2023).

Fresh edible mushrooms undergo various stages from farm to store and finally to consumers, affecting their quality and shelf life (Schill et al., 2021). However, compared to other fresh produce, data on the microbiological profile of fresh edible mushrooms remain limited. Because of their susceptibility to rapid quality deterioration and short shelf life, implementing effective preservation techniques and advanced assessment technologies is essential to mitigate these challenges (Schill et al., 2021; Meng et al., 2024). The growing body of research on microbial communities highlights the importance of examining preservation methods and their effects on the microbiome of fresh edible mushrooms. Culture-independent techniques have demonstrated that, in natural environments, fungi-associated microbiota colonize mycelial surfaces and surrounding substrates (Halsey et al., 2016). Mushrooms are linked to different microbes, and the levels of coliforms, mesophilic aerobes, psychrophilic aerobes, molds, and yeasts differ among various mushroom species (Schill et al., 2021; Meng et al., 2024).

Although fungi have coexisted and interacted with bacteria since early fungal evolution, our understanding of these interactions remains limited. Bacterial communities strongly influence the microenvironment within and around fungal hyphae, and can prevent many macrofungal species from forming fruiting bodies under sterile conditions (Noble et al., 2009). Unsurprisingly, bacterial-fungal interactions significantly affect the productivity of commercial mushroom farms (Li et al., 2017; Carrasco and Preston, 2020). Fungi can also modify the microbiome of their growing environment, which, in turn, influences their development and fruiting (Zhou et al., 2017). Ultimately, this bidirectional interaction shapes both the fungal host and its associated microorganisms, which play a crucial role in mushroom productivity (Carrasco and Preston, 2020; Partida-Martínez, 2017).

Growing interest in the microbiomes of edible mushrooms, combined with rapid advances in sequencing technologies, underscores the need for an updated synthesis of how microbial communities influence mushroom quality and safety throughout the postharvest chain. Despite expanding global production and rising consumer demand for minimally processed foods, major knowledge gaps remain regarding microbial succession, host–microbe interactions, and environmental factors that shape spoilage dynamics—areas that existing reviews often address only partially or in isolation. Additionally, the urgency of reducing postharvest losses and adopting sustainable, microbiome-informed preservation strategies underscores the need for a timely reassessment of the current evidence. By addressing these gaps, the present review provides a timely assessment of (i) microbial contamination and microbiome composition and function across edible mushroom species, and (ii) environmental and processing factors shaping microbial dynamics, and key determinants of microbial behavior, while (iii) integrating bibliometric analyses to highlight emerging research trends, persistent gaps, and opportunities to improve food safety and quality through targeted intervention strategies. It further evaluates current and emerging preservation and contamination-prevention strategies through a microbiome-centered lens. Through this integrated perspective, the review highlights opportunities to leverage microbial ecology to develop more sustainable, effective approaches that support food safety, reduce spoilage, and strengthen global food security.

## 2 Microbiome composition and microbial contamination of fresh edible mushrooms

Mushrooms are primarily composed of water, with moisture content typically exceeding 85–90% of fresh weight. They continue to respire and metabolize actively even after harvest, thereby accelerating moisture loss, enzymatic browning, and texture deterioration. Fresh mushrooms have delicate, soft tissues, lack a protective cuticle, and are therefore highly susceptible to mechanical damage and microbial contamination (Huo et al., 2023). Microbial contamination refers to the proliferation of undesirable microorganisms beyond established safety or quality thresholds during cultivation, harvest, processing, storage, or distribution. Additionally, microbial spoilage and anaerobic respiration are closely associated with the development of unpleasant odors and discoloration in edible mushrooms (Carrasco and Preston, 2020). Studying the microbial load of fresh mushrooms can help prevent contamination and potentially extend their shelf life. An overview of recent studies addressing microbial associations with edible mushrooms is summarized in Table 2.

**Table 2:** Recent studies on microbial association and microbiome composition of fresh edible mushrooms.

Mushroom species studied	Microbiome composition (dominant/key genera/phyla)	Specific contaminants/pathogens isolated/detected	Country/region	References
*Agaricus bisporus* (Button mushroom)	The abundance of *Pseudomonas* on the surface of fresh *A. bisporus* was 22.8%.	*Pseudomonas tolaasii* was isolated and found to cause serious spoilage of *A. bisporus* in pathogenicity tests.	China	Hou et al. (2023)
*Agaricus bisporus* and *Pleurotus eryngii*	Six slow-growing, gram-positive bacterial strains resembled members of Microbacteriaceae (Actinobacteria).	A new bacterial disease, bacterial brown pit, was caused by *Mycetocola* sp. on the cut cap surfaces of the mushrooms.	Iran	Hamidizade et al. (2020)
*Agaricus bisporus* (Button mushroom from local marketplaces)	Bacterial strains resembling the members of Enterobacteriaceae were the most predominant group of pathogenic agents isolated in Iran.	*Ewingella americana* strains initiated symptoms ranging from faint discoloration to dark brown and blotch on mushroom caps.	Iran	Hamidizade et al. (2023)
*Lentinus edodes, Pleurotus ostreatus, and Hypsizygus marmoreus*	Proteobacteria was the predominant phylum in the edible mushroom samples during storage.	The enteric bacteria cluster was a predominant bacterial population along with *Pseudomonas*, *Burkholderia*, *Lactococcus*, *Sphingobacterium*, and *Stenotrophomonas*.	China	Xia et al. (2024)
*Flammulina filiformis* (Golden needle mushroom)	The dominant bacterial phyla in fresh *F. filiformis* were *Proteobacteria, Bacteroidetes, Firmicutes, Actinobacteria, and Cyanobacteria.*	*Pseudomonas, Lactobacillus, Acinetobacter, Flavobacterium, and Phyllobacterium Ewingella*, *Serratia*, and *Pseudomonas* became the main dominant genera in packaged *F. filiformis* after postharvest storage.	East Asia and China	Fang et al. (2021) and Liu et al. (2024)
*Pleurotus ostreatus, Pleurotus eryngii,* and *Lentinula edodes* (Retail/Stored)	*Pseudomonas fluorescens* species complex was the most abundant species/subgroup isolated from all sample types, comprising 54.7% of isolates. Other bacteria of *Enterobacteriaceae, Lactic acid bacteria* and *Bacillus cereus* were also present	*Ewingella americana* was detected in 45.2% of the total mushroom samples analyzed.	Microbiological studies carried out with mushrooms obtained from Austria, Germany, Poland, and South Korea	Schill et al. (2021)
*Pleurotus eryngii* (King oyster mushroom)	The prevalence rate of *Listeria monocytogenes* in the production facility environment was 14.3%.	*Listeria monocytogenes* was detected in 13.3% of the mushroom samples analyzed from production plants.	China	Xu et al. (2023)
*Pleurotus Tuoliensis* (commonly known as Bailinggu)	*Pseudomonadata, Bacteriodata, Bacillota*	Strains of *Bacillus* sp. were identified to enhance the growth of *Pleurotus tuoliensis* in natural habitat	Western China	Luo et al. (2025)
*A. bisporus* (Commercially cultivable)	*Trichoderma aggressivum f. aggressivum* and *Pseudomonas tolaasii*	Microbial assays showing the effect of passaged casing on the pathogens *Pseudomonas tolasii* (causing brown blotch) and *T aggressivum* f. *aggressivum* (responsible for green mold).	Study carried out in Mushroom facility in Pennsylvania State University, USA	O’Connor et al. (2024)
*Agaricus bisporus* (Button mushroom)	Causal agent of ginger blotch *Pseudomonas gingeri,* *P. tolaasii, P. reactans* and *P. costantinii P. yamanorum,* *P. edaphica, P. salomonii*	*Pseudomonas* sp., *Dyadobacter* sp., *Pedobacter* sp., and *Flavobacterium* sp. were other species present that suppressed disease causing pathogen	Study carried out in Mushroom growing facility of Wageningen University, Netherlands, Western Europe	Taparia et al. (2021b) and Taparia et al. (2021a)
*A*. *bisporus*	1st report on the fungal infectant *Lecanicillium fungicola* on white-button mushroom	*Lecanicillium fungicola*	Northern Vietnam	Thao et al. (2020)
*Agaricus bisporus* (Stored)	*Pedobacter* and *Flavobacterium* grow vigorously on the eighth day of cold storage while *Pseudomonas*, *Ewingella* and *Chryseobacterium* were present on later period of storage. *Pseudomonas* most prevalent genus, 87.01% in fresh samples during cold storage.	*P. azotoformans*, *P. proteolytica*, *P. tolaasii*, and *P. brenneri* were amongst the specific *Pseudomonas* species detected in retail samples. *Pseudomonas tolaasii* caused brown blotch upon inoculation on the cap surface.	China	Qiu et al. (2019)

### 2.1 Microbiome composition study of edible mushrooms

The mushroom microbiome encompasses the entire microbial community associated with the host and its substrate across developmental and postharvest stages, including taxa that are beneficial, neutral, or potentially harmful. Understanding this microbiome requires tools to study the underlying bacterial–fungal–mushroom interactions using diverse microbiological approaches (Venturini et al., 2011; Carrasco and Preston, 2020). Despite their long co-existing evolutionary history, these interactions remain insufficiently characterized. Although both culture-dependent and culture-independent methods have been applied (Salar and Aneja, 2007; Longley et al., 2019), traditional techniques only identify a limited fraction of microbial diversity due to inherent biases. In contrast, Next-Generation Sequencing (NGS), paired with advanced bioinformatics, has become a powerful method that dramatically expands the exploration of microbial diversity in different environmental niches. It enables detailed profiling and uncovers previously uncultured taxa that are relevant to mushroom cultivation efficiency (Halsey et al., 2016; Agrawal et al., 2015). High-throughput data generation is primarily achieved through parallel sequencing of nucleic acid libraries (Satam et al., 2023). Since mushrooms develop in microbe-rich substrates and maintain close interaction with bacteria and fungi throughout their life cycle, NGS enables comprehensive identification of associated microbial taxa, including rare and previously unculturable species (Thai et al., 2022). Techniques such as 16S rRNA and Internal Transcribed Spacer (ITS) sequencing for fungi, whole-genome sequencing, shotgun metagenomics, and metatranscriptomics allow researchers not only to identify microbial taxa but also to explore their functional potential in greater detail (Tedersoo et al., 2022; Giusti et al., 2024). These approaches have revealed complex interaction networks, including bacteria that stimulate mycelial growth, microbes that enhance nutrient mineralization within the substrate, and antagonistic species responsible for diseases such as green mold (*Trichoderma* spp.) or bacterial blotch (Giusti et al., 2024; Ali et al., 2022; Ajis et al., 2024).

With declining sequencing costs, NGS has become an accessible and effective way to analyze microbial communities, typically categorized into operational taxonomic units (OTUs) or amplicon sequence variants (ASVs), depending on the analytical approach, supported by more advanced bioinformatics pipelines and data analysis tools (Tedersoo et al., 2022; Doytchinov and Dimov, 2022). The generated OTUs or ASVs are processed through various pipelines and tools that filter or denoise them, resulting in readable outputs. These generated outputs offer high-resolution insights into microbial diversity, taxonomic structure, and functional gene potential within complex substrates. They also enable robust characterization of their roles in fermentation and help establish microbial indicators for assessing the quality and stability of mushroom cultivation substrates and culture media (Giusti et al., 2024; Nam et al., 2023; Suwannarach et al., 2022). Despite these advances, mushroom-derived food products remain relatively understudied in terms of microbial characterization, even with the availability of standard NGS methodologies. To date, only a limited number of studies have applied metabarcoding approaches to authenticate edible mushroom species in commercially available products in the United States—such as dried and powdered mushrooms, soups, pasta sauces, and flavor enhancers. This gap may be attributed not only to the historically modest market share of mushrooms but also to regulatory, methodological, and cost-related constraints that have limited routine molecular authentication in processed foods (Giusti et al., 2024).

### 2.2 Culture-independent studies on bacterial community composition of mushrooms

**Figure 1:**
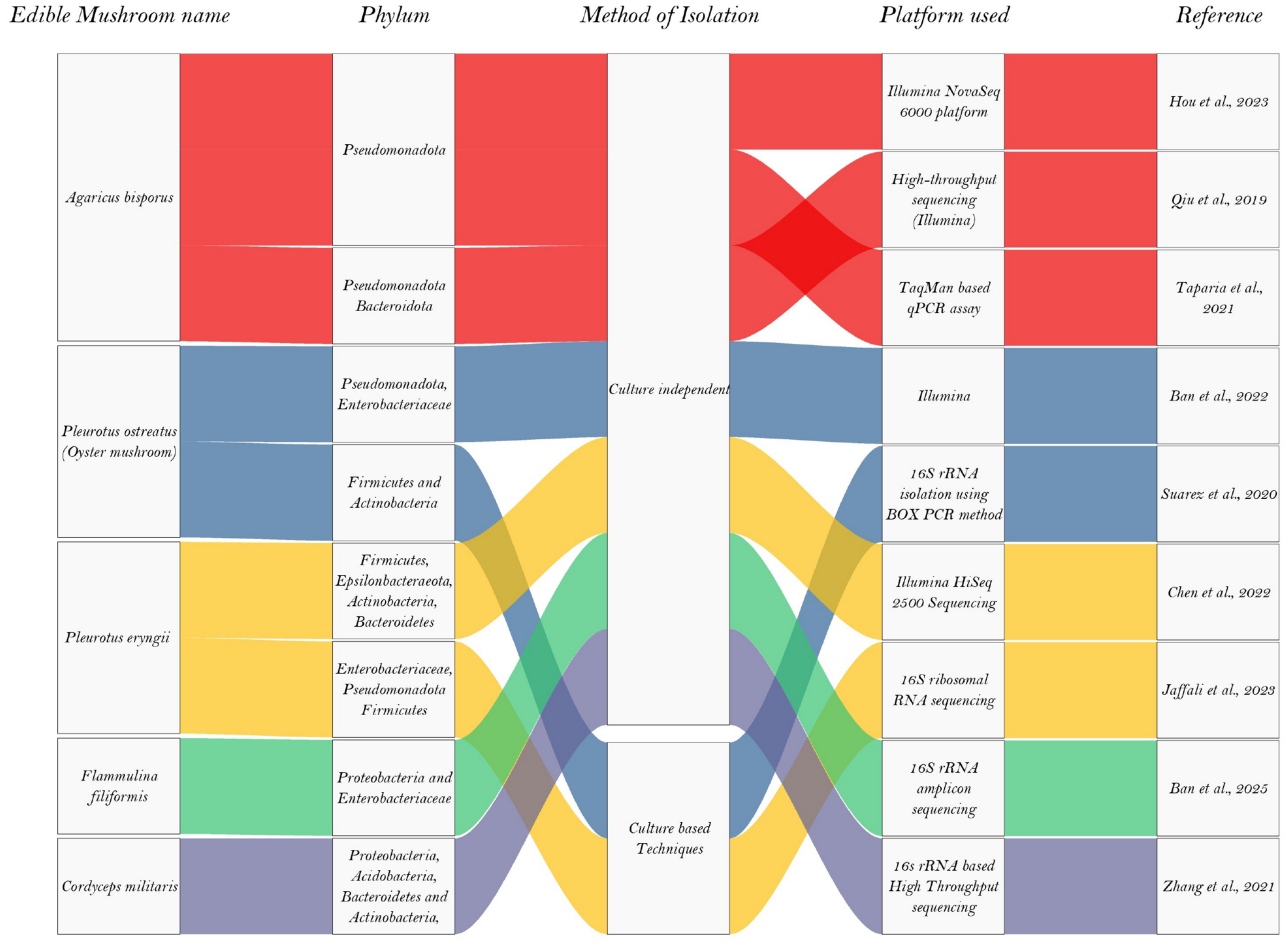
Graphical representation of the most abundant phylum in contaminating edible mushrooms in recent years.

Researchers have conducted metagenomic and amplicon-based studies on various mushroom species at different growth stages to determine how bacterial communities influence mushroom quality after harvest and during storage. This section summarizes culture-independent investigations of bacterial community composition across major edible mushroom species. The associated relationships are visualized in a graphical format as a Sankey plot (Figure 1) showing abundances of microbial phylum which is also summarized in Table 3, highlighting the sequencing platforms and analytical approaches used in each study.

**Table 3:** Most abundant bacterial phylum in contaminating edible mushrooms in recent years.

Edible mushrooms	Most reported species of bacteria	Phylum	Method of isolation	Platform used	References
*Agaricus bisporus*	*Pseudomonas tolaasii*	Pseudomonadota	Culture-independent	Illumina NovaSeq 6,000 platform	Hou et al. (2023)
	*Ewingella, Pseudomonas, Chryseobacterium*	Pseudomonadota Bacteroidota	Culture-independent	High-throughput sequencing (Illumina) and MALDI-TOF mass spectrometry fingerprinting	Qiu et al. (2019)
	*Pseudomonas salomonii*	Pseudomonadota	Culture-independent	TaqMan based qPCR assay	Taparia et al. (2021b)
*Pleurotus ostreatus* (Oyster mushroom)	*Enterobacteria*, *Lactococcus*, *Pseudomonas*, and *Weissella*	Pseudomonadota, Enterobacteriaceae	Culture-Independent	Illumina	Ban et al. (2025)
	*Bacillus, Paenibacillus and Micromonospora*	Firmicutes and Actinobacteria	Culture based Techniques	16S rRNA isolation using BOX PCR method	Suarez et al. (2020)
*Pleurotus eryngii*	*Acinetobacter, Escherichia-Shigella, Corynebacterium, Achromobacter, Cutibacterium, and Brevibacterium*	Firmicutes, Epsilonbacteraeota, Actinobacteria, Bacteroidetes	Culture-Independent	Illumina HiSeq 2,500 Sequencing	Chen et al. (2022)
	*Bacillus subtilis,* *B. tequilensis,* *B. cereus, Ewingella americana, and Klebsiella grimontii*	*Enterobacteriaceae, Pseudomonadota Firmicutes*	Culture based Techniques	16S ribosomal RNA sequencing	Jaffali et al. (2023)
*Flammulina filiformis*	*Lactococcus, Leuconostoc, Pseudomonas, and Staphylococcus*	Proteobacteria and Enterobacteriaceae	Culture-Independent	16S rRNA amplicon sequencing	Ban et al. (2025)
*Cordyceps militaris*		Proteobacteria, Acidobacteria, Bacteroidetes and Actinobacteria	Culture-Independent	16 s rRNA based High Throughput sequencing	Zhang et al. (2021)

#### 2.2.1 Bacterial community studies on *Agaricus bisporus*

*Agaricus bisporus* is one of the most common edible mushrooms, and it is the most widely cultivated and consumed worldwide. It is characterized by its white appearance and high-moisture content (>90%) (Tarlak, 2020). Because of its nutrient-rich composition and high water activity, it is particularly susceptible to microbial colonization, which may lead to spoilage and quality deterioration. Therefor*e*, studying the microbial community associated with *A*. *bisporus* is essential for developing preservation techniques that enable long-term storage.

The cultivation cycle of *A. bisporus* serves as a valuable model system because it relies heavily on ecological interactions with diverse microorganisms present in compost and casing layers. Bioreactor simulations of compost pasteurization (57 °C for 6 h, 60 °C for 2 h, and 68 °C for 2 h) have been evaluated using 16S rRNA gene sequencing to assess their impact on bacterial community dynamics (Vieira and Pecchia, 2018). High-pasteurization temperature reduced bacterial alpha diversity and increased the relative abundance of Bacillales, which was associated with elevated ammonia emissions. However, overall bacterial structure (beta diversity) remained relatively stable across treatments. Excessive pasteurization temperature significantly inhibited *Agaricus bisporus* mycelial growth, suggesting that maintaining balanced microbial communities is critical for optimal cultivation (Vieira and Pecchia, 2018).

Commercial cultivation of *A. bisporus,* particularly prevalent in European and Western Countries (Shamugam and Kertesz, 2023), typically utilizes fermented and pasteurized substrates composed of lignocellulosic agricultural residues such as wheat straw, cattle manure, or poultry manure (Suwannarach et al., 2022; Vieira et al., 2023). During composting, bacteria and fungi synergistically decompose complex organic matter, generating a selective substrate that supports mushroom growth. Several studies have demonstrated that microbial community dynamics during composting are essential for substrate degradation and successful mycelial colonization (Kertesz and Thai, 2018; Vieira and Pecchia, 2022). Both the biological and physicochemical properties of the substrate undergo substantial transformations, converting raw materials into a selective medium that promotes mushroom growth (Vieira et al., 2023). Although physical and chemical changes during substrate preparation are well documented, microbial succession patterns during fermentation remain comparatively less characterized. These microbial communities continuously respond to variations in nutrient availability, temperature, oxygen concentration, moisture, and the carbon-to-nitrogen ratio—factors that strongly influence community composition and substrate quality (Thai et al., 2022).

Traditionally, researchers have relied on pure culture methods to identify spoilage bacteria associated with *A. bisporu*s. For instance, Munsch et al. (2002) discovered a novel species, *Pseudomonas costantinii,* while Taparia et al. (2020) isolated known spoilage bacteria, including *P. gingeri*, *P. tolaasii*, *P. reactans*, and *P. costantinii,* from the mushroom caps, along with several previously unknown spoilage bacteria. However, culture-based approaches capture only a fraction of the total bacterial diversity, as many spoilage microorganisms are not readily cultivable on standard laboratory media. Consequently, direct DNA extraction combined with high-throughput sequencing has become essential for the comprehensive characterization of bacterial communities in *A. bisporus* samples. For instance, one study employed Illumina HiSeq 2500sequencing to analyze bacterial diversity on button mushrooms during cold storage. Bacteria detected at later storage stages were further characterized using 16S rRNA gene sequencing and MALDI-TOF mass spectrometry (Matrix-Assisted Laser Desorption/Ionization Time-of-Flight) to rapidly identify spoilage-associated taxa and inform shelf-life-tending strategies (Qiu et al., 2019). Taxonomic analysis revealed *Pseudomonas* as the dominant genus across storage conditions, with *Ewingella* and *Chryseobacterium* emerging during prolonged storage (Qiu et al., 2019). Another study investigated temporal shifts in bacterial community dynamics during storage of *A. bisporus,* using Illumina 6,000 sequencing to analyze taxonomic variation and predict metabolic functions (Hou et al., 2023). Samples exhibiting black spot symptoms were further analyzed to identify pathogenic bacteria isolated with spoilage (Hou et al., 2023). Metagenomic and 16S rRNA sequencing approaches have also identified thermophilic actinomycetes as key contributors to lignocellulose degradation during composting, highlighting their potential biotechnological relevance. Overall, research on bacterial community dynamics in *A*. *bisporus* cultivation has primarily focused on complex microbial processes during substrate preparation and composting. In contrast, comparatively few studies have examined bacterial succession throughout the entire cultivation cycle—from compost preparation to postharvest storage—representing an important gap in current knowledge.

#### 2.2.2 Bacterial community studies on *Pleurotus* sp.

*Pleurotus* sp., particularly *Pleurotus ostreatus* (oyster mushroom), are widely cultivated edible mushrooms, especially popular in Asian countries (Shamugam and Kertesz, 2023). Studies on *Pleurotus ostreatus* cultivation reveal a dynamic succession of bacterial taxa, strongly influenced by growth stage and substrate type, that persist after harvesting (Vieira and Pecchia, 2022; Banfi et al., 2021). Characterizing beneficial bacteria across cultivation substrate types and elucidating fungal–bacterial interactions are essential for improving production efficiency and mushroom quality (Chen et al., 2022). During substrate colonization and fruiting, relative abundances of *Thermus* spp. and *Actinobacteria* decrease, whereas *Bacillales* and *Halomonas* spp. become more prevalent (Banfi et al., 2021). This shift has been associated with declining temperature, a change in pH from ~7 to ~5, and the secretion of enzymes, such as laccases, by *P. ostreatus* hyphae (Banfi et al., 2021). The microbial community, including microbes and their metabolites, plays a protective role against competing fungi without hindering *P. ostreatus* growth. Genera such as *Pseudomonas* may promote fungal growth, whereas certain *Bacillus* spp. may suppress competing molds, enhance carbohydrate utilization, and reduce contamination during cultivation (Banfi et al., 2021; Liu et al., 2022).

Detailed analyses of bacterial communities in substrates such as wheat straw and short-composting corn cob highlight the importance of microbial activity in lignocellulose degradation and its direct association with mushroom yield. Overall, these findings emphasize the critical role of bacterial-fungal interactions in optimizing *P. ostreatus* production and minimizing contamination (Banfi et al., 2021). Using 454 pyrosequencing, the native substrate microbiota for *Pleurotus* cultivation was shown to include *Paenibacillus* and *Bacillus* species, while dominant fungal sequences were assigned to *Wallemia* and *Verticillium* spp. (Jablonský et al., 2016). A Sanger sequencing-based 16S rRNA gene study assessing the microbial quality of cultivated *Pleurotus ostreatus* and *Pleurotus eryngii* identified members of *Enterobacteriaceae*, *Pseudomonadaceae*, Lactobacilli, and yeasts. Culture-dependent analyses further detected *Bacillus cereus* during late storage at 4 °C, and reported the presence of *Salmonella* spp. and *Listeria monocytogenes* in stored samples (Schill et al., 2021). Despite these findings, most NGS studies on *Pleurotus* spp. have focused primarily on substrate preparation and compost microbiology. Relatively few investigations have examined bacterial community dynamics directly associated with the mushroom tissues during harvest and storage, representing a significant gap in post-harvest microbiome research.

#### 2.2.3 Bacterial community studies on *Lentinula edodes*

Shiitake mushrooms (*Lentinula edodes*) have gained popularity for their nutritional value, culinary properties, and health-promoting properties. Over the past decade, its cultivation has expanded rapidly, making it a major contributor to the global mushroom industry (Gil-Ramírez et al., 2016). *The growth and productivity of Lentinula edodes* involve complex interactions with bacterial communities that influence substrate degradation, mycelial growth, and final mushroom quality (Suwannarach et al., 2022; Huang et al., 2022). However, this species is characterized by a short shelf life, high microbial load, and rapid postharvest deterioration (Schill et al., 2021; Tejedor-Calvo et al., 2020).

Cultivation of *Lentinula edodes* on composted sawdust has been shown to shorten the time to brown-film formation and to enhance mycelial growth and crude polysaccharide content compared with fresh sawdust. Transcriptomic, qRT-PCR, and proteomic analyses revealed significant alterations in metabolic pathways, including RNA polymerase activity, starch and sucrose metabolism, and tyrosine metabolism (Huang et al., 2022). In particular, genes involved in aromatic amino acid metabolism were upregulated in the composted sawdust substrate, suggesting that composting modifies substrate chemistry and microbial composition, enhances nutrient availability, and fungal development through lignin and cellulose degradation (Huang et al., 2022). Studies indicate that using composted sawdust can shorten the time to brown film formation and increase mycelial growth rate, possibly due to higher nitrogen levels in the composted substrate. Although enzyme activities involved in lignocellulosic breakdown may not exhibit statistically significant differences between composted and uncomposted substrates, changes in substrate quality and microbial communities collectively support the growth and polysaccharide content of *L*. *edodes*.

Postharvest studies further demonstrate the role of bacterial communities in shiitake deterioration. Tejedor-Calvo et al. (2020) monitored physico-chemical, microbiological, and sensory parameters of shiitake mushrooms under three storage conditions until complete carpophores degradation (Tejedor-Calvo et al., 2020). A total of 48 strains were identified, of which *E. americana*, *Burkholderia* sp., *Serratia* sp., *Rahnella* sp., and some *Pseudomonas* strains showed degradation abilities of shiitake carpophores (Tejedor-Calvo et al., 2020). The biodeterioration potential of these isolates increased during storage, with cap slices showing greater susceptibility to damage than intact cap cuticle (Li et al., 2022). Moreover, bacterial isolates from *L. edodes* demonstrated pronounced pathogenic effects on the mushroom tissues (Schill et al., 2021; Tejedor-Calvo et al., 2020).

Microbial profiling of the brown film of *L. edodes* stage has also identified potential bacterial biomarkers capable of distinguishing cultivation stages (Tejedor-Calvo et al., 2020). Moreover, inoculating compost substrates with beneficial microbes can enhance biological efficiency and mushroom quality, underscoring the importance of microbiome management in *L. edodes* cultivation (Schill et al., 2021; Suwannarach et al., 2022; Tejedor-Calvo et al., 2020). Nevertheless, comprehensive studies examining microbial succession during postharvest shelf life remain limited.

#### 2.2.4 Bacterial community studies on *Flammulina filiformis*

*Flammulina filiformis* (commonly known as enoki mushroom) is a widely cultivated edible and medicinal mushroom in East Asia (Yan et al., 2019; Fang et al., 2021). Global demand has increased due to its applications in traditional and functional foods, nutraceuticals, and as a source of bioactive compounds in pharmaceutical development. Advances in substrate formulation, cultivation technology, and quality control have improved yield, disease resistance, and production sustainability (Jayaraman et al., 2024; Sharma et al., 2024).

High-throughput 16S rRNA gene amplicon sequencing has been employed to characterize bacterial communities associated with *F. filiformis* across cultivation environments and storage conditions. These studies have identified dominant bacterial phyla, including *Proteobacteria*, *Firmicutes*, *Bacteroidetes*, *Actinobacteria*, and *Cyanobacteria*, as well as genera such as *Pseudomonas*, *Lactobacillus*, *Acinetobacter*, *Flavobacterium*, and *Phyllobacterium* (Liu et al., 2024). Regional variation has been observed in the relative abundance of *Pseudomonas* and *Lactobacillus* populations. Pathogenic species, including *Pseudomonas tolaasii*, *Ewingella americana*, and *Janthinobacterium lividum,* have been confirmed as causing agents of black rot and light brown rot in *F. filiformis* (Liu et al., 2024). On the other hand, beneficial taxa can enhance nutrient availability, strengthen host defenses, and suppress pathogens, underscoring the importance of microbiome management to optimize yield, improve product quality, and promote sustainable cultivation (Fang et al., 2021; Liu et al., 2024).

#### 2.2.5 Research gaps and future directions

Most existing studies on edible mushroom microbiomes emphasize substrate optimization, cultivation parameters, and fungal physiology. In contrast, the taxonomic composition, functional roles, and ecological dynamics of associated bacterial communities, particularly during harvest, storage, and distribution, remain insufficiently characterized. Furthermore, relatively few integrate advanced omics tools—such as shotgun metagenomics, metatranscriptomics, or metabolomics —to assess functional activity across cultivation and postharvest stages. Collectively, these gaps highlight the need for high-resolution, stage-specific microbiome analyses that combine multi-omics approaches with cultivation and storage data. Such integrated strategies will clarify microbial community structure, functional potential, and ecological contributions, thereby enabling evidence-based interventions to enhance productivity, product quality, and sustainability in edible mushroom systems.

### 2.3 Sources of contamination

Insufficient post-harvest management and improper storage conditions can result in contamination by microorganisms originating from the air, soil, or surrounding environment (Saikia and Bora, 2023; Thakur et al., 2025). These microorganisms may accelerate mushroom spoilage and deterioration and, in some cases, pose health risks to consumers. Spoilage occurs when microorganisms degrade cellular structures, leading to loss of turgor pressure, cellular collapse, and tissue maceration rather than merely reducing the vesicle size (Castellanos-Reyes et al., 2021). Additionally, poor handling practices, mechanical damage to mushroom surfaces, and high moisture levels promote bacterial proliferation. After packing at processing facilities, mushrooms should be transported and stored under controlled temperature and humidity conditions, as retail display at room temperature can significantly accelerate microbial growth and quality loss (Fang et al., 2021; Quevedo et al., 2016).

The primary source of microbial contamination in mushrooms is contact with the cultivation substrate. Mushrooms are typically grown on carbon-rich, nutrient-dense materials such as compost, manure, lignocellulosic residues, and agricultural byproducts, which provide an ideal ecological niche for diverse microbial populations. Inadequate pasteurization or sterilization may allow pathogenic bacteria such as *E. coli* and *Salmonella*, as well as opportunistic mold and pathogenic fungi, to persist in the production system and compete with or colonize developing mycelia (Zhang et al., 2014; Suarez et al., 2020). Environmental factors such as contaminated irrigation water, insufficient air filtration, improperly sanitized harvesting tools, and inadequate worker hygiene further increase the contamination risk. Soil-borne microorganisms may persist throughout the growth cycle because mushrooms absorb water and nutrients directly from the substrate, underscoring the importance of maintaining strict hygiene and environmental control during cultivation (Carrasco and Preston, 2020).

## 3 Microbial contaminants responsible for the spoilage of edible mushrooms

Edible mushrooms harbor diverse endogenous microbiomes that play crucial roles in growth, nutrient cycling, and physiological development during cultivation. These microbial communities often exert beneficial or neutral effects under controlled conditions. However, under favorable environmental or post-harvest conditions, certain members of the microbiota may shift to opportunistic or pathogenic behavior, contributing to tissue degradation and spoilage (Deveau et al., 2018). Therefore, comprehensive characterization of microbial diversity and community composition across cultivation and postharvest stages is essential for understanding spoilage dynamics.

Understanding microbial contaminants in edible mushrooms requires a comprehensive assessment of microbial diversity and community composition, as well as elucidation of the roles these microorganisms play across various stages of mushroom cultivation. Substrate-specific and casing layer interactions strongly influence disease development. The casing layer, typically composed of nutrient-poor peat applied over compost, triggers the transformation from vegetative mycelial growth to reproductive fruiting. Although nutritionally limited, casing provides an ecological niche for a diverse microbiome that plays a role in fructification and disease expression. Comparative studies evaluating black peat, blonde peat, and a 50:50 mixture under artificial inoculation with *Lecanicillium fungicola* and *Mycogone perniciosa*, alongside uninoculated controls, and agronomical practices such as ruffling, demonstrated that black and mixed peat produced higher yields of healthy mushrooms. However, none of these treatments significantly suppressed disease under high pathogen pressure. Metagenomic analyses revealed that the casing microbiota strongly shapes the bacterial community of harvested mushrooms, with nearly 50% compositional similarity. The detection of *L. fungicola* in asymptomatic harvested mushrooms suggests that casing-associated microbial communities may exert antagonistic effects only at low initial inoculum levels (Carrasco et al., 2019).

Among major microbial diseases, bacterial blotch, caused by *Pseudomonas tolaasii*, and green mold, caused by *Trichoderma aggressivum*, are particularly destructive in cultivated mushrooms, including *Agaricus bisporus.* These diseases result in brown discoloration, surface lesions, and reduced productivity. The casing layer often serves as primary reservoir for these pathogens, and disease severity correlates with microbial population structure within the casing.

Studies involving “passaged casing” (casing soil reused across multiple cropping cycles) have demonstrated the complex ecological role of accumulated microbiota. Passaged casing, developed through 10 successive cropping cycles by mixing primordia-stage material with fresh casing, was evaluated in controlled container trials (n = 8 replicates per treatment) following inoculation with *P. tolaasii* (blotch) and *T. aggressivum* (green mold). Results indicated that passaged casing reduced blotch incidence but increased susceptibility to green mold, highlighting the dual and context-dependent role of casing microbiota in disease modulation (O’Connor et al., 2024).

Several bacterial species cause significant quality and yield losses; *E. americana* has been reported to cause browning and necrosis in the stipe of *Agaricus bisporus*. It is found in the stipe of *Agaricus bisporus* and is increasingly recognized as a multifaceted pathogen linked to diseases in edible mushrooms and also responsible for spoilage (Taparia et al., 2020; Taparia et al., 2021b; Hamidizade et al., 2023). Fruiting bodies may develop brown or yellow-to-dark brown lesions due to toxin production by *Pseudomonas torulosa* and related species (Qiu et al., 2019; Taparia et al., 2021b). Brown blotch disease is caused by several *Pseudomonas* species, including recently identified *P. salomonii* and *P. edaphica,* which are phylogenetically related to the blotch-causing taxa *P. constantinii* and *P. fluorescens* (Taparia et al., 2020; Taparia et al., 2021b). The genus *Pseudomonas* is widely regarded as a primary contributor to spoilage in white button mushrooms (*Agaricus bisporus*). In fresh samples, its relative abundance was 22.8%, increasing to 68.7% after 6 days of storage and reaching 84.5% by day 12. Other genera responsible for spoilage, like *Serratia*, have also been identified, with their occurrence increasing from 0.4% early on to 10.6% by the eighteenth day. Both *Pseudomonas* and *Serratia* are psychrotrophic bacteria that can proliferate at refrigeration temperatures, making them major contributors to post-harvest deterioration. Consequently, members of the phylum Proteobacteria play a dominant role in mushroom spoilage dynamics (Hou et al., 2023).

To reduce microbial contamination, implementing effective contamination control strategies is essential (Grimm et al., 2024). Also, a deeper understanding of the microbial community structure and function, especially the balance between microorganisms that suppress disease and those that promote it, is critical for sustainable mushroom production. Future research should prioritize mechanistic studies of mushroom–microbe interaction, integrating ecological, molecular, and postharvest perspectives to enhance production efficiency, disease control, and product quality.

## 4 Factors influencing microbial contamination and microbiome composition

A dynamic interplay between intrinsic biological factors and extrinsic environmental conditions governs microbial contamination and the composition of the microbiome in cultivated edible mushrooms. The major influencing factors are described below:

### 4.1 Growth substrate

The growth substrate used in mushroom cultivation plays a crucial role in shaping microbial community structure, which, in turn, influences production efficiency and final product quality. Substrate formulation, typically composed of agricultural or agro-residual residue, must meet the specific nutritional requirements of the cultivated mushroom species (Suwannarach et al., 2022). Mushrooms can be broadly classified by their decomposition strategy. Primary decomposers, such as *Lentinula* and *Pleurotus* species, grow directly on relatively undecomposed lignocellulosic materials, often after sterilization. In contrast, secondary decomposers, such as *Agaricus* species, need partially decomposed, nutrient-rich substrates created through controlled aerobic solid-state fermentation (composting). Composting induces significant physical, chemical, and biological transformations, notably the critical C/N ratio, which must be closely monitored. For example, the ideal C/N ratio for *A. bisporus* is approximately 19/1, while *Lentinula edodes* needs a ratio of 30–35:1 (Cueva et al., 2002). Accordingly, the selection of raw materials and treatment processes (sterilization or composting) directly influences the initial microbial community and the selective pressures imposed during cultivation. The complexity of the mushroom production system results in substantial microbial succession throughout the life cycle, largely driven by substrate composition and the degree of decomposition. Research on *A. bisporus* cultivation indicates that the dominant bacterial phyla in compost and casing are generally Proteobacteria, Firmicutes, Bacteroidetes, and Actinobacteria. Typically, Firmicutes and Actinobacteria predominate in compost and casing soil, whereas Bacteroidetes are more frequently detected in mushroom caps (Mcgee et al., 2017). Additionally, during the thermophilic composting phases, critical for secondary decomposers, thermophilic fungi such as *Mycothermus thermophilus, Talaromyces thermophilus,* and *Thermomyces lanuginosus* proliferate and produce lignocellulosic enzymes that facilitate biomass degradation (Vieira et al., 2023; Martins et al., 2013). These microbial consortia are essential for lignocellulose breakdown, nitrogen transformation, and the regulation of biochemical conditions conducive to fruiting body formation.

Fungal metabolites’ activity further drives shifts in bacterial composition, diversity, and functional potential. For example, during the cultivation of the medicinal fungus *Ganoderma lucidum* on a substrate containing cottonseed hull, wheat bran, corn flour, and gypsum, the bacterial community was dominated by Proteobacteria and Firmicutes, accounting for 80.12–95.93% of the detected taxa across growth stages. However, the substrate’s physical and chemical properties, including pH, moisture content, and concentration of total nitrogen, phosphorus, and potassium, changed significantly during fungus development. Notably, bacterial richness and diversity peaked during the elongation stage of *G. lucidum,* coinciding with the minimum levels of nitrogen, phosphorus, and potassium (Zhang et al., 2018). This pattern likely reflects intensified nutrient competition and metabolic demand during rapid fungal growth, illustrating how fungal physiology alters the substrate environment and drives bacterial community restructuring.

### 4.2 Storage conditions

Storage conditions are critical in determining the shelf life of fresh mushrooms, as their high moisture content (~90%) and absence of a protective cuticular layer increase susceptibility to microbial contamination and rapid spoilage (Burton and Noble, 1993). Fresh mushrooms sampled from retail markets in Spain exhibit initial microbial loads ranging from 10^4.4^ to 10^9^ CFU/g, particularly in samples with high water activity (Khalloufi et al., 2000). Fresh *Lentinula edodes* (shiitake) displayed water activity values of approximately 0.99, which correlated with the presence of aerobic mesophiles, *Bacillus* sp., yeast, and coliforms (Kim et al., 2016). Key bacterial spoilage genera include *Pseudomonas*, *Enterobacter*, and *Rahnella*, with *Pseudomonas* often being the most prevalent spoilage organism (Cao et al., 2024).

Storage temperature and packing atmosphere significantly influence microbial proliferation and microbiome composition. Refrigeration is essential for maintaining quality and safety. At ambient temperature, mushrooms typically remain marketable for only 1–3 days, whereas refrigeration can extend shelf life to 5–7 days (Jiang, 2013). In studies on *A. bisporus* stored at elevated temperatures (17 °C and 25 °C), packaging type significantly affected microbial growth (González-Fandos et al., 2000). Mushrooms packed in perforated PVC film maintained higher oxygen levels, favouring aerobic spoilage organisms. After five days, *Pseudomonas* populations increased to 9.3 log CFU g^−1^ at 17 °C and 9.7 log CFU g^−1^ at 25 °C, representing increases from initial levels (6.6 log CFU g^−1^) (González-Fandos et al., 2000). Mold contamination was detected in 80% of samples within two days. In contrast, non-perforated PVC film created a modified atmosphere (~7% CO_2_ and <1% O_2_). The reduced oxygen environment suppressed aerobic bacteria growth, resulting in lower *Pseudomonas* counts after five days (8.8 log CFU g^−1^ at 17 °C and 8.1 log CFU g^−1^ at 25 °C). However, this anaerobic environment enabled the detection of anaerobic spores (~2 log CFU g − 1 at 25 °C) in non-perforated film packages (González-Fandos et al., 2000). Commercial fresh mushroom samples have been found to contain pathogenic bacteria, including a low prevalence of *Campylobacter jejuni* and *Staphylococcus* spp., with only four isolates identified as *Staphylococcus aureus* (Venturini et al., *2011*). For *Pleurotus ostreatus* (oyster mushrooms), substrate composition also influences postharvest contamination levels. Mushrooms grown on a mixture of olive pruning residues, spent coffee grounds, and wheat straw maintained contamination levels below 5.0 log CFU/g during the first day at 20 °C. Under refrigeration at 4 °C, mushrooms packed in polyethylene or vacuum polypropylene bags maintained low contamination counts for seven days. Importantly, mushrooms grown on optimized substrate mixtures and stored at 4 °C exhibited contamination levels of only 4.2–4.9 log CFU/g even by day 15. The generally acceptable contamination limit for packed mushrooms stored at 4 °C (below 8.1 log CFU/g) was extended to approximately 22 days under optimized conditions (Abou Fayssal et al., 2023). These findings demonstrate that effective preservation depends on synergistic control of temperature, atmospheric composition, packaging permeability, and initial substrate-associated microbiota.

### 4.3 Geographical location

The microbiome composition of fresh mushrooms is strongly influenced by geographical location, which shapes both microbial diversity and community structure. Variations in climate, soil characteristics, and local microbial communities affect how microbial communities form around mushrooms. Temperature, rainfall, and soil pH are key factors that influence the abundance and distribution of bacteria and fungi. In addition to regional agricultural practices and post-harvest handling procedures, these microbial patterns are further shaped by these factors. Understanding these geographical influences is therefore crucial for improving mushroom quality, safety, and shelf life.

The mushroom microbiome, often referred to as Mushroom-Inhabiting Bacteria (MIB), is structured by complex interactions among the fungal host, environmental conditions, and local microbial communities. At a global scale, host phylogeny appears to exert a greater influence on MIB community composition than macro-environmental variables. Accordingly, Gohar et al. (2022) found that host identity explains approximately 4.7% of the variation in bacterial communities, whereas biome type accounts for 1.7%, mean annual temperature for 0.6%, and mean annual precipitation for 0.1%. Geographical location plays a role in shaping MIB, as demonstrated by a global dataset of 330 fruiting bodies collected from 90 sites across 31 countries. These samples were obtained from regions including Northern and Eastern Europe (Estonia and Sweden), North America (USA), South and Central America (Chile, Argentina, and Guyana), Africa (Cameroon and South Africa), and Asia (Pakistan and Laos), representing diverse biomes such as tundra, temperate forests, tropical forests, and savannas. Although geographical distance influenced bacterial community composition, it had limited effects on overall diversity, and MIB diversity did not follow a clear latitudinal gradient. For example, Actinobacteria and Firmicutes were less abundant in savanna regions compared to tropical and temperate forests (Gohar et al., 2022). Overall, MIB diversity shows weak correlations with large-scale environmental gradients such as latitude, temperature, rainfall, soil pH, or organic carbon content, highlighting the importance role of host-related factors in shaping global microbiome patterns.

In contrast, at the local and regional scales, environmental variables become more influential (Gohar et al., 2022). In boreal forest ecosystems, soil pH has been identified as a key driver of bacterial community structure associated with mushrooms, second only to fungal taxonomic identity. Permutational multivariate analysis of variance (PERMANOVA) showed that soil pH accounted for 6.20% (adjusted R^2^) of community variation, while fungal order accounted for 9.27%, with both factors acting independently. The local microbial community serves as the primary source of MIB, with about 41% of bacterial OTUs detected in mushroom fruiting bodies also present in nearby soil. However, colonization is highly selective. Mushroom-associated microbiomes typically differ from bulk soil communities, often exhibiting reduced relative abundance of Acidobacteria and Actinobacteria, while being enriched in Proteobacteria, likely due to the carbon-rich environment provided by fungal tissues. Evidence further suggests the existence of a global core mushroom microbiome, comprising recurrent genera such as *Halomonas, Serratia, Bacillus, Cutibacterium*, *Bradyrhizobium,* and *Burkholderia* (Pent et al., 2017). The repeated detection of these taxa across geographically distinct regions suggests conserved ecological roles in nutrient cycling, host interactions, or fruiting body development.

## 5 Microbial contamination and microbiome composition of edible mushrooms: a bibliometrics analysis

To better understand microbial contaminants involved in edible mushroom spoilage and the factors influencing microbiome composition, it is essential to assess the current research landscape. This review addresses this need through a bibliometric analysis of publication trends, keyword co-occurrence, and global research patterns.

### 5.1 Methodology for bibliometric analysis

The Web of Science (WoS) database was systematically searched using relevant keywords related to *microbiome composition* and *microbial contamination in fresh mushrooms*. The search covered 10-year period (2016–2025) to capture recent advances and emerging trends. A total of 4,409 publications, including research articles, review papers, and early access documents, were retrieved. Bibliometric analysis was performed using VOSviewer (Koley et al., 2025; Gupta et al., 2024) for network visualization and RStudio (Bhat et al., 2023; Gupta et al., 2026) for statistical processing and trend analysis.

### 5.2 Keywords networking

**Figure 2:**
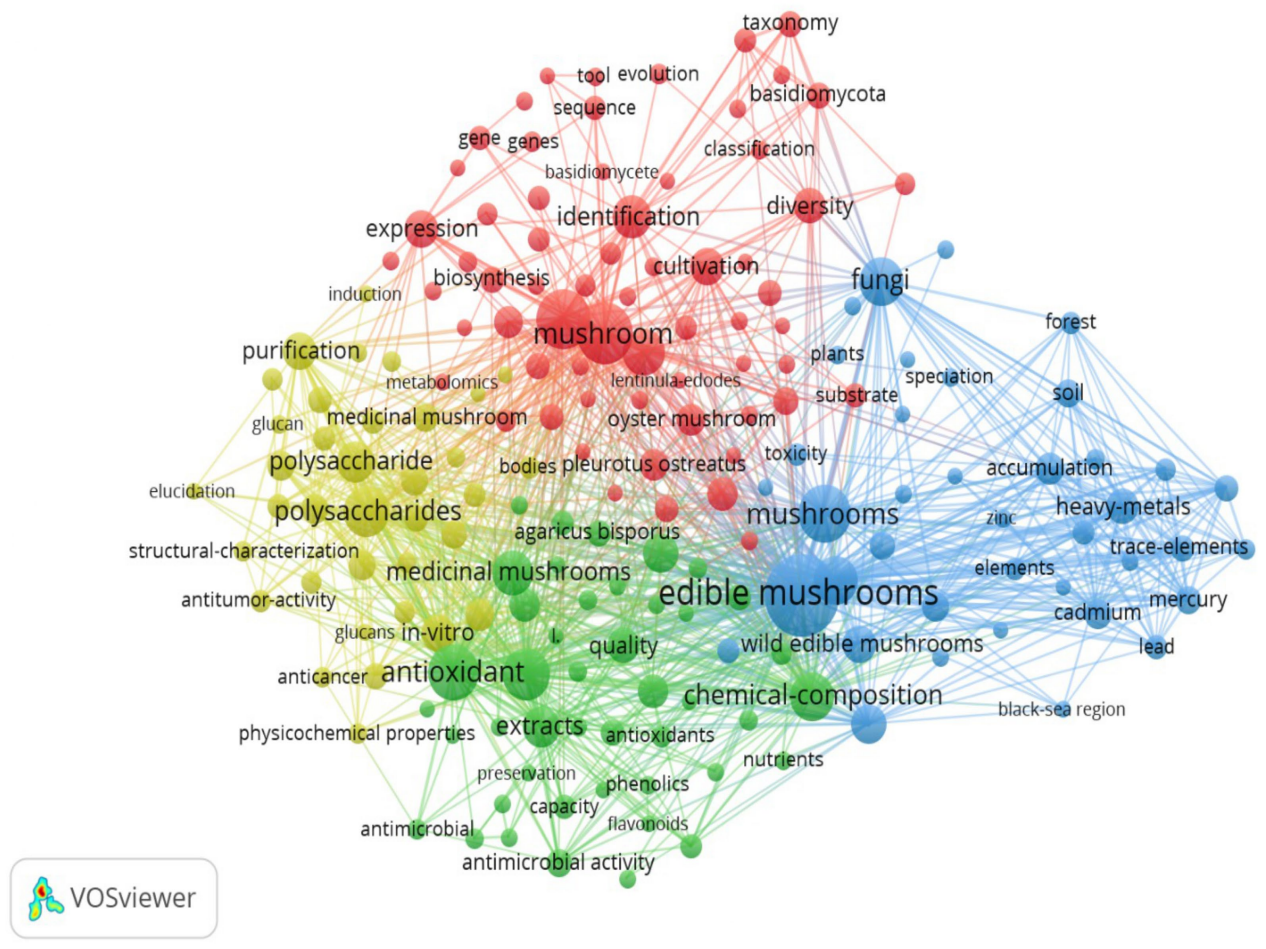
Keyword co-occurrence network map.

Exported records were analyzed in VOSviewer to map a keyword co-occurrence network (Figure 2). The analysis identified 183 items grouped into four thematic clusters, representing the major research themes related to mushroom microbiology. A total of 8,788 links and a total link strength of 37,874 were observed, indicating strong interconnections among keywords and a high degree of research coherence across subtopics. The association strength normalization method was applied to build the co-occurrence network, ensuring balanced visualization and accurate clustering (Gupta et al., 2024; Koley et al., 2023).

The resulting network revealed four interrelated research domains.

- Cluster 1 (Red – Taxonomy, Genetics, and Cultivation): The cluster focuses on fungal taxonomy, molecular identification, and cultivation biology. It emphasizes genetic diversity, molecular classification, and gene expression analysis in major genera such as *Pleurotus*, *Agaricus*, and *Lentinula*. Research in this domain explores how genetic and physiological traits influence microbial community structure, contamination susceptibility, and product safety.
- Cluster 2 (Green – Bioactive Compounds and Functional Properties): The cluster focuses on bioactive compounds and their functional roles, including polysaccharides, phenolics, flavonoids, and antioxidants. These compounds exhibit antimicrobial and antioxidant activities, potentially contributing to the natural defense system against spoilage organisms. Research also addresses extraction techniques and purification strategies, linking metabolite composition to both nutritional quality and extended shelf-life.
- Cluster 3 (Blue – Environmental Contamination and Elemental Accumulation): The cluster addresses environmental pollutants and heavy metal accumulation in mushrooms, including cadmium, lead, mercury, and zinc. Research highlights how soil composition, substrate quality, and environmental exposure influence microbial diversity and food safety, potential altering in native microbiome.
- Cluster 4 (Yellow – Medicinal Mushrooms and Biochemical Characterization): The cluster stresses structural characterization of bioactive compounds, metabolomic profiling, and functional analysis of polysaccharides and glucans. It links microbial interactions with therapeutic potential, safety assessment, and processing technologies in medicinal mushroom production.

Collectively, these clusters illustrate the multidisciplinary nature of mushroom microbiology research, integrating genetics, microbial ecology, bioactivity, environment science, food safety, and functional biochemistry. This structured knowledge framework provides a foundation for advancing contamination control strategies, enhancing nutritional value, and optimizing post-harvest management.

### 5.3 Publication trends

A decade-long analysis (2015–2024) revealed a steady, significant increase in publications on microbiome composition and microbial contamination in fresh mushrooms. In 2015, 311 publications were recorded, marking the early stages of intensified research interest. Moderate growth occurred between 2017 and 2019, followed by a pronounced rise after 2020, aligned with advancements in microbial sequencing and food safety research. The highest annual output was observed in 2024, with 661 publications, more than double the number reported in 2015. This upward trend indicates the expanding scientific focus on mushroom-associated microbiomes, food safety, and post-harvest contamination management.

**Figure 3:**
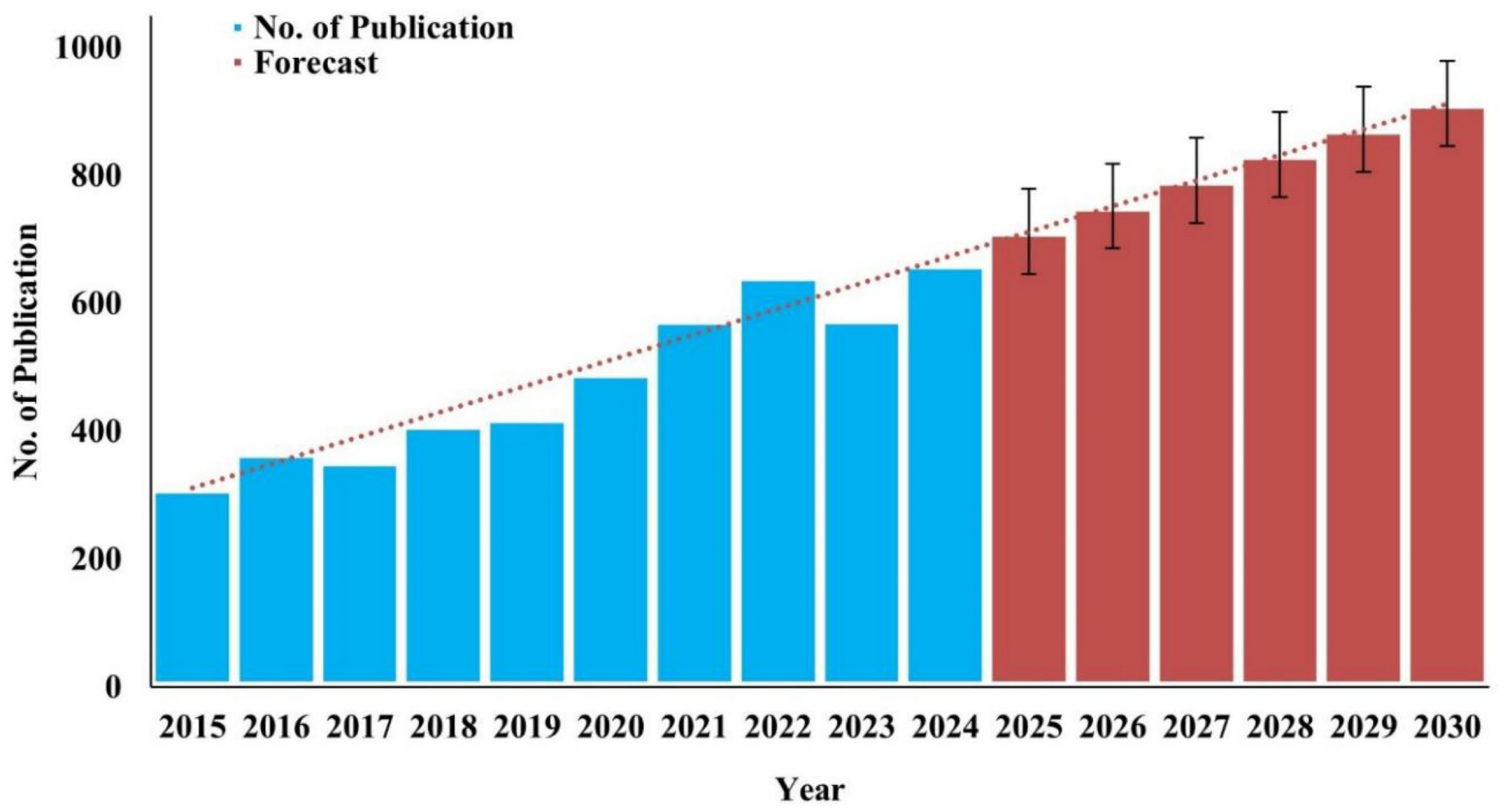
Publication trend over the past decade with forecasted growth in the coming years.

Trend-based forecasting suggests continued growth in research publications, potentially reaching approximately 912 publications by 2030. The projected increase underscores the sustained relevance of microbiome research at the intersection of food safety, environmental health, and sustainable agriculture (Figure 3).

### 5.4 Global scientific contribution

The country-wise analysis shows substantial global engagement in research on the microbiome composition and microbial contamination of edible mushrooms, with several nations leading the field. China ranks first with 1,827 publications, reflecting its strong emphasis on food safety, microbial ecology, agricultural biotechnology, and metagenomics. This high research output likely corresponds to the country’s large-scale mushroom production industry and advanced genomic research infrastructure. India ranks second with 285 publications, focusing on post-harvest spoilage, microbial safety, and the nutritional microbiome, particularly under tropical climatic conditions. The United States is closely followed by 275 publications, contributing significantly to food microbiology, metagenomics, and industrial hygiene. European and East Asian countries also demonstrate strong research engagement. Poland (253), Japan (179), and Mexico (170) also make significant contributions, likely linked to their established mushroom cultivation sectors and interest in sustainable production systems.

**Figure 4:**
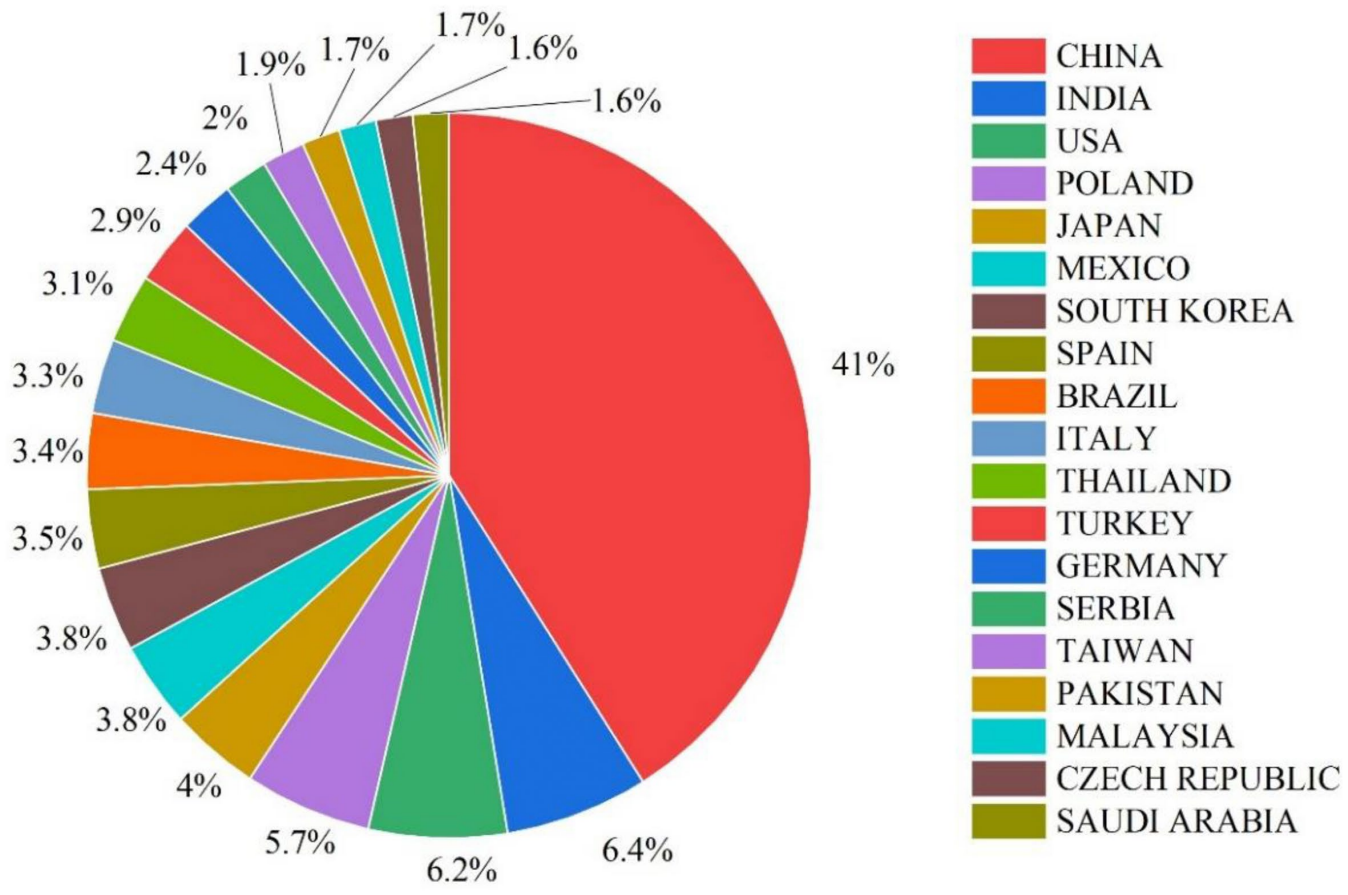
Global contribution and country-wise distribution of publications in this research field.

Additional contributions from South Korea (168), Spain (Thao et al., 2020), Brazil (Bhambri et al., 2022), and Italy (Chowdhury et al., 2015) further highlight the multidisciplinary nature of fungal microbiome research, including applied, environmental, and clinical perspectives. Emerging participation from Thailand, Turkey, Germany, and Serbia reflects the expansion of regional research networks. Meanwhile, countries such as Pakistan, Malaysia, the Czech Republic, and Saudi Arabia show smaller but increasing outputs, indicating growing awareness of the safety of microbiomes and their biotechnological potential in mushroom production (Figure 4). Collectively, the global distribution of publications underscores the increasing international recognition of mushroom microbiome research as a critical component of food safety, sustainable agriculture, and functional food development.

## 6 Control and prevention of microbial contaminants

Mushroom farming is a labor-intensive, biologically sensitive process that requires strict contamination control to ensure product quality and yield. Contamination may occur at various stages, including substrate preparation, spawning, cultivation, harvesting, post-harvest handling, storage, and transportation (Suwannarach et al., 2022). Effective contamination control begins during substrate preparation—standard preventive measures, including pasteurization or sterilization to eliminate spores and other competing microbes. Sterilization is commonly achieved through autoclaving, whereas pasteurization may involve controlled heat application, hot water, steam exposure, or approved chemical treatments (Grimm et al., 2024; Gowda and Manvi, 2019). Sterilization via autoclaving using saturated steam at 121 °C under 15 psi pressure for 15–30 min effectively eliminates most vegetative cells and spores, including resilient green mold spores. In contrast, pasteurization employs lower thermal intensities (e.g., hot water at 63–70 °C for 1.5 h or hot air at 75–100 °C for 3 h), which reduce but do not eradicate the microbial population, leaving residual microbiota that may influence subsequent cultivation (Grimm et al., 2024; Bellettini et al., 2019).

Maintaining optimal environmental conditions during cultivation is equally critical. A moderate temperature that favours mycelial colonization enables the cultivated fungus to rapidly dominate the substrate, thereby suppressing competing molds and bacteria (Hu et al., 2023). The casing layer applied over colonized compost plays a crucial role in fruiting body development and productivity. However, excessive moisture in the casing layer can promote the germination and proliferation of pathogenic fungi (Navarro et al., 2021). Even when substrates are properly pasteurized, the use of contaminated irrigation water may reintroduce pathogens, such as *Trichoderma,* leading to significant crop loss (Ajis et al., 2024).

### 6.1 Managing pre-harvest contamination of mushrooms

#### 6.1.1 Spawn contamination

Spawn, or the “seed” of mushrooms, functions as the main inoculum for cultivation and is essential for successful growth (Goltaphe and Purjam, 2003). The quality of spawns directly influences the efficiency of mycelial colonization and the final yield. Selection of an appropriate spawn carrier (commonly cereal grains) is essential for producing high-quality inoculum and ensuring uniform mycelial development (Balakrishnan and Nair, 1997). Fungal and bacterial contaminations remain one of the most significant challenges in spawn production (Bhargav et al., 2023). *Trichoderma harzianum* is considered one of the most destructive contaminants, particularly affecting *Pleurotus pulmonarius* and *Pleurotus ostreatus* during spawn and substrate colonization (Biswas, 2016). Substrate types and grain preparation methods, including the number of boiling cycles, greatly influence contamination rates (Gupta et al., 2020). Proper soaking, boiling, drying, and sterilization procedures are critical for minimizing microbial survival. Irradiation treatment at a dose of 3.8 megarads has been reported to effectively destroy spores within grains and associated materials (e.g., glassware or polypropylene bags) while maintaining high spawn quality and nutrient reserves for button mushroom production (Dong et al., 2026).

#### 6.1.2 Mushroom hut/house sanitation and fumigation

Strict hygiene and sanitation practices in mushroom-growing houses are essential for preventing disease (Jacob et al., 2025; Aminuzzaman et al., 2022). Fumigation alone is insufficient without prior thorough cleaning and removal of organic debris. All tools, equipment, and cultivation materials must be disinfected before use. Additionally, personnel movement should be controlled to minimize the introduction of fungal spores and other contaminants into the growing environment (Aminuzzaman et al., 2022). Footbaths, protective clothing, and restricted access protocols are recommended to reduce pathogen spread. Integrated sanitation strategies—combining environmental cleaning, chemical disinfection, controlled ventilation, and biosecurity measures—are necessary to effectively manage microbial contamination in mushroom production systems (Sharma et al., 2007).

### 6.2 Disinfection of substrates

#### 6.2.1 Substrate disinfection by chemicals

Heat-based sterilization methods can be technically demanding and costly in large-scale mushroom production systems. Chemical sterilization eliminates microbial contaminants from substrates and is easier to manage and more cost-effective, making it suitable for large-scale substrates. Commonly used chemical disinfectants in edible mushroom cultivation include formaldehyde, carbendazim, limestone, calcium hydroxide, and hydrogen peroxide (Saritha and Pandey, 2010). These agents reduce microbial load by disrupting cellular membranes, denaturing proteins, or altering substrate pH, creating unfavorable conditions for contaminants. Disinfection with bleaching agents is also effective in reducing contamination by spraying from mushroom initiation through the end of the crop cycle, using chlorine dioxide and sodium hypochlorite at various concentrations and contact times with the substrate (Szumigaj-Tarnowska et al., 2012; Han et al., 2001). Fungicidal treatments such as benomyl, a combination of carbendazim and blitox, and thiram are highly effective and fully inhibit competing molds infecting oyster mushrooms in both *in vitro* and *in vivo* conditions (Sharma et al., 2007). However, the routine use of chemical fungicides must be carefully regulated due to potential environmental persistence, resistance development, and concerns about residues in food products.

#### 6.2.2 Pasteurization and sterilization of substrate

Substrate pasteurization process is an inexpensive method that can be carried out at different temperatures and durations, using steam or hot water, thereby eliminating a variety of pathogens (Balasubramanya and Kathe, 1996; Sánchez, 2010). In high-temperature pasteurization, vapors from a chamber or tunnel containing the substrates are used at around 80 °C for 2 h. Alternatively, substrates can be soaked in water for 24 h, then pasteurized for 2 h, with excess water drained before mixing for final use. After cooling, the substrates are inoculated with spawn (Nasir et al., 2021). Thermal pasteurization is the best disinfection method because it eliminates the major competing microorganisms from the substrates. This method helps retain major nutrients and allows beneficial microorganisms to survive. There are several types of pasteurization methods, but scalding substrates at 80 °C for short times (<30 min) has been found to be more effective, and the use of tunnels in substrate processing is the most suitable equipment (González et al., 2022).

Steam pasteurization is also commonly used to prepare substrates for edible mushroom cultivation worldwide. This method also helps eliminate microbial contaminants and enhance mycelial growth (Sharma et al., 2007; O'Neill et al., 2015; Atila, 2016). The pasteurization of the substrate at 65 °C for 6–8 h is effective for softening the texture of the substrate and eradicating mesophilic microorganisms such as *Trichoderma* spp., *Coprinus* spp., *Penicillium* and *Aspergillus*, but mould spores are destroyed at temperatures above 80 °C (Choi, 2004).

Substrate sterilization is widely used in developed countries and is considered adequate in mushroom cultivation (Kim et al., 2013; Ryu et al., 2015). However, it is not an effective method on its own, as it kills both beneficial and harmful organisms in the substrate. In contrast, pasteurization selectively kills only temperature-sensitive microorganisms, creating enough space for mushroom mycelium to spread and establish a colony (Mejía and Albertó, 2013). Using sterilization is not mandatory for all species; for some, substrate pasteurization alone is sufficient to minimize damage to mycelial development and yield caused by pathogenic organisms (Sánchez, 2010). Several alternative methods for preparing substrates exist, although most research favors the axenic cultivation of mushrooms on substrates sterilized by autoclaving (Dias, 2010; da Silva et al., 2012). While axenic cultivation of mushrooms is effective, its commercialization is hindered by high costs. Recent research on contamination methods highlights a gap and emphasizes the need for more focus to enhance cost-effectiveness and sustainability.

#### 6.2.3 Biological control agents for substrate contamination

Biocontrol agents such as *Bacillus velezensis* QST 713 (Serenade^®^) are registered for use in mushroom cultivation, offering a targeted, sustainable approach to control microparasitic pathogens that cause substantial crop losses (Jacob et al., 2025). Similarly, *Trichoderma* spp. has been shown to inhibit harmful fungi, such as *Lecanicillium fungicola,* by competing for nutrients and space in the substrate (Berendsen et al., 2010; Preston et al., 2019). The biocontrol agent Mycostop^®^ (*Streptomyces griseoviridis*) is effective against *Lecanicillium fungicola* in cropping experiments (Preston et al., 2019). The use of antagonistic bacteria as biocontrol agents is an effective strategy for managing bacterial blotch diseases caused by *Pseudomonas tolaasii* and other *Pseudomonas* specie*s* (Largeteau and Savoie, 2010). Lactonases derived from bacterial strains have been shown to effectively control green mold infestations in mushroom farms (Krupke et al., 2003). In biocontrol, nematodes such as *Steinernema* spp. are used to control fly larvae by releasing symbiotic bacteria (*Xenorhabdus* spp. or *Photorhabdus* spp.), which subsequently destroy the infested individuals (Anderson et al., 2021). Plant phenolics and essential oil extracts are also employed to prevent mycelial growth of *Trichoderma harzianum* under both *in vitro* and *in vivo* conditions in certain mushroom species (Hatvani et al., 2012; Tanhaeian et al., 2020). The *in vivo* application of grape seed extract containing naringin, a strong antibacterial agent, during pre- and post-sprouting of mushrooms was reported to be effective in preventing bacterial infections and subsequent degradation of *Pleurotus eryngii* mushroom (Murgia et al., 2024). Essential oils and medicinal plant extracts from *Bunium persicum*, *Lippia citriodora*, *Mentha piperita*, and *Thymus vulgaris* successfully prevent the growth of *Trichoderma harzianum* (Tanhaeian et al., *2020*).

### 6.3 Managing *post-harvest* contamination of mushrooms

#### 6.3.1 Treatments and storage of edible mushrooms

Post-harvest characteristics of fresh edible mushrooms include shrinkage, weight loss, softening, discoloration, and reduced levels of flavor compounds and nutrients. These have a limited shelf life, lasting only 1–3 days at room temperature and 5–7 days when refrigerated (Feng et al., 2023; Jiang, 2013). During harvesting, transport, storage, and retail, their quality is affected, presenting significant challenges for postharvest preservation and commercial distribution (Zhong et al., 2022). Mechanical damage and invasion of spoilage microbes can further cause decay and degeneration (Castellanos-Reyes et al., 2021; Zheng et al., 2023). Recent treatments using organically derived ergothioneine extract show promise as a bioactive compound for reducing oxidative browning and improving post-harvest quality in *A. bisporus*. These treatments help maintain higher levels of phenolics and ascorbic acid in mushrooms (Cao et al., 2024; Qian et al., 2021). Additionally, immersing *A*. *bisporus* in exogenous *γ*-aminobutyric acid enhances phenylalanine and ammonia-lyase activity, effectively delaying browning during refrigeration (Qian et al., 2021; Shekari et al., 2021). Electrolyzed water, produced by electrolyzing neutral salt solutions, contains active oxygen species that provide strong disinfection, bacteriostatic, and cleaning effects. It effectively destroys microbial cells and their internal structures by generating reactive oxygen species and altering local pH, which inhibits spoilage-causing microbes on edible mushroom surfaces (Aday, 2016).

#### 6.3.2 Maintenance of the dispatching area and transport vehicle hygiene

Keeping the mushroom harvest area clean helps preserve their quality and reduces the risk of cross-contamination that can cause rapid spoilage. Maintaining hygiene in the dispatching area, delivery facilities, and transport by following proper cleaning and disinfection procedures is important for mushroom preservation. Mushroom transport vehicles should not exceed 12 °C, with an ideal temperature range of 2 °C to 4 °C to prevent spoilage. Operators should follow safe handling practices in the dispatching room and during transport to prevent tray damage and microbial contamination (Pardo et al., 2011).

#### 6.3.3 Preservation technologies

Choosing the right packaging materials is crucial for maintaining the stability and preserving the shelf life of fresh mushrooms. Current commercial mushroom packaging often uses materials like polyethylene, polyvinyl chloride, and polypropylene. According to some research, the use of micro-perforated film improves permeability, and microporous membranes help maintain ideal color and reduce the formation of odor compounds, as seen in *A*. *bisporus* (Pogorzelska-Nowicka et al., 2020; Cheng et al., 2022). Emerging food preservation technologies encompass a range of innovative approaches, such as (i) *cold plasma treatment* uses an electric field to ionize a sealed gas, generating sterilizing plasma—the fourth state of matter; (ii) *electrostatic field treatments*, including high-voltage (HVEF) and low-voltage (LVEF) systems, are non-thermal approaches used with refrigeration to help prolong shelf life; (iii) *active packaging* incorporates functional components that release antimicrobial, antioxidant, moisture-regulating, or odor-controlling agents; (iv) *edible coatings* involve applying a thin, food-grade layer to the product surface through dipping or spraying; (v) *antimicrobial photodynamic therapy* uses light-activated photosensitizers to generate reactive oxygen species capable of destroying microorganisms; (vi) *natural preservatives* derived from biological sources act as antibacterial, antioxidant, anti-browning, and anti-aging agents when foods are dipped, sprayed, or fumigated; (vii) *genetic editing* enhances preservation by modifying genetic traits to prolong shelf life (Cao et al., 2024; Abou Fayssal et al., 2023; Qian et al., 2021; Pogorzelska-Nowicka et al., 2020). Additionally, techniques like ultrasound treatment, ultrasound combined with irradiation, air-ion treatment, pulse light, and pulsed electric field are effective in preserving mushrooms (Cao et al., 2024; Abou Fayssal et al., 2023). These techniques help extend the shelf life of mushrooms, which is particularly important for maintaining their nutritional content, including vitamins, minerals, and bioactive compounds.

## 7 Role of edible mushroom in food security, and rural livelihood opportunities: case studies

Edible mushrooms contribute significantly to food security and offer sustainable livelihood opportunities in rural areas, particularly in resource-limited settings. By utilizing lignocellulosic agro-waste such as paddy straw, sugarcane bagasse, and sawdust as substrates, mushroom production converts materials that would otherwise be discarded or burnt into highly nutritious food (Cunha Zied et al., 2020). From a rural development perspective, mushroom cultivation is particularly attractive due to its low land requirement, modest capital investment, and limited labor demand compared to conventional agriculture (Galano and Santos, 2024). Production systems can be implemented on a small scale, indoors or vertically, making them accessible to marginalized communities (Galano and Santos, 2024).

Importantly, mushroom farming has been recognized as a gender-inclusive enterprise (Mayanja and Tipi, 2017). It offers income-generating opportunities for women and self-help groups (SHGs), enabling them to earn income without transgressing prevailing social norms or household responsibilities (Domingo, 2025). Case studies from Sri Lanka demonstrate that income generated through mushroom cultivation enhances women’s financial autonomy and decision-making capacity, contributing to socioeconomic empowerment (Jayasinghe, 2024). Edible mushrooms are nutritionally rich, containing high-quality protein, dietary fiber, essential vitamins, and minerals (El-Ramady et al., 2022). Their inclusion in the diet supports food and nutritional security, especially in vulnerable populations affected by malnutrition and micronutrient deficiencies. In several regions, wild edible mushroom harvesting also contributes to livelihood resilience. For example, in Tanzania, the collection and sale of wild edible mushrooms provide both a nutritional food source and a rapid local capital-come opportunity (Tibuhwa, 2013). Numerous global case studies further demonstrate the contribution of different mushroom species to food security, rural income generation, and community resilience. Table 4 summarizes the documented roles of various edible mushrooms in enhancing nutrition, supporting livelihoods, and strengthening local economies.

**Table 4:** Edible mushroom, food security, and rural livelihood opportunities: case studies.

Case study (country and references)	Mushroom focus/species	Food security contribution	Rural livelihood opportunity	Key livelihood mechanism/case
Chhattisgarh, India (Thakur et al., 2025)	Major species cultivated commercially are white button (*Agaricus bisporus*), oyster (*Pleurotus* spp.), paddy straw (*Volvariella volvacea*), milky mushroom (*Calocybe indica*), and shiitake. Nine production units in Chhattisgarh mainly produce *Pleurotus* species and *Calocybe indica*.	Mushrooms are an excellent source of nutrition and quality carbohydrates, rich in protein, vitamins, and minerals. Cultivation is a viable option to ensure protein-rich nourishment and income security for the rural population by utilizing agro-waste. Mushrooms help fight anemia, undernutrition, and malnutrition.	Mushroom cultivation is a low-cost, highly remunerative enterprise involving less space and expenditure, allowing the recycling of agro-waste. It can double or triple the income within a year. It supports cash income of a regular nature, improving the socio-economic standards of rural people.	The Indira Gandhi Krishi Vishwavidyalaya (IGKV) and Krishi Vigyan Kendras (KVKs) provide extensive training to rural women and youths. Under tripartite agreements with SRLM/MGNREGS (2016–2017), 1741 youths/women were trained in mushroom production technology to adopt it as an income-generating activity in Chhattisgarh.
Bangladesh (Dey et al., 2024)	Focus is on sustainable mushroom farming using agri-waste as substrates. Oyster mushrooms are commonly produced.	Mushroom farming offers a sustainable solution to food security challenges stemming from inadequate and imbalanced diets. Mushrooms provide a necessary source of protein, vitamins, and minerals.	The industry offers job opportunities, particularly for women, reducing unemployment. Mushroom farming is a profitable enterprise for smallholder farmers due to flexibility in farm size and labor availability.	Strategic planning (using TOPSIS) identified that the top-ranking strategy for sustainable growth is to secure funding and provide more accessible loan options. The involvement of family labor, especially women, augments profit margins.
Kegalle District, Sri Lanka (Jayasinghe, 2024)	Commonly cultivated varieties include American Oyster, Bhutan Oyster, Button, Paddy Straw, Abalone, and Milky White Mushrooms. *Pleurotus* spp. are highly prominent.	Mushrooms contribute to household nutrition and food security through direct consumption, offering an affordable source of high-quality protein and essential micronutrients. Consumption enhances dietary diversity, especially where access to animal protein is limited.	Mushroom farming is an accessible agribusiness venture, particularly for women-led households, due to low land/capital requirements and a short production cycle (approx. 2.5 months). It provides a stable, year-round source of income.	Women’s economic independence from mushroom income translates into greater negotiating power and autonomy in household decision-making regarding expenditures and resources (Agency/Achievements, based on Kabeer’s framework).
Rural Tanzania (Wild Harvest) (Tibuhwa, 2013)	Focuses on Wild Edible Mushrooms (WEM) harvested from forests, including genera such as *Termitomyces*, *Cantharellus*, *Lactarius*, *Rusulla*, *Amanita*, and *Boletus*.	Wild edible mushrooms contain essential compounds like proteins, vitamins, minerals, and fatty acids, which provide healthy food for rural dwellers. Harvesting and eating WEM contributes largely to food security and improved livelihood.	WEM collection is an economic alternative for disadvantaged groups, including old people, women, and children, requiring only energy spent gathering. A collector can generate an average of $400–$900 annually.	WEM collection is dominated by women (76.25%) in rural areas, who often gain social recognition as “mama uyoga” (mother of mushroom). It is valued for its contribution to subsistence income generation.

## 8 Discussion and conclusion

Microbial contamination is a major factor limiting the quality, shelf life, and safety of fresh edible mushrooms (Carrasco and Preston, 2020; Chen et al., 2022; Ban et al., 2025; Jacob et al., 2025). While certain microorganisms aid in breaking down complex substrates and support fruiting body development, others cause post-harvest contamination and spoilage. Consequently, gaining a deeper understanding of the mushroom-associated microbiota is vital for better contamination management, post-harvest preservation, and the consistent global product quality. Importantly, contamination-related losses remain a significant economic challenge in commercial mushroom farming worldwide. Exploring the roles of beneficial microbiota opens new avenues for biological control strategies and for reducing dependence on chemical use. Approaches guided by microbiome analysis could greatly improve mushroom yield stability, shelf life, and safety. Therefore, incorporating microbiome knowledge into commercial mushroom cultivation is increasingly essential for sustainable industry growth.

Advanced NGS studies have significantly enhanced our understanding of mushroom-associated microbiomes, uncovering complex, dynamic microbial communities that shape beneficial interactions and spoilage across widely cultivated species. However, significant gaps still exist in understanding how functional interactions, pathogen ecology, and environmental and handling conditions affect microbial dynamics. Future research should leverage advances in NGS, including improved resolution and analytical frameworks, in combination with culture-based approaches to more precisely elucidate microbial functions and ecological interactions (Giusti et al., 2024). Microbial dynamics during storage, transport, and retail display must be carefully monitored, as these stages significantly impact shelf life and economic losses. Developing standardized sampling protocols, specialized databases of mushroom-associated microbes, and predictive contamination models will improve the reproducibility and practical use of research outcomes.

The bibliometric analysis presented in this review confirms the growing global research interest in the mushroom microbiome and contamination management; yet critical ambiguities persist regarding key mechanisms and effective contamination mitigation approaches. Although *Agaricus bisporus* is the most extensively cultivated and studied species, research on the commercially important edible mushrooms remains comparatively limited. This underscores the need for broader investigations across diverse species to support market diversification and long-term sustainability.

Beyond microbial management, edible mushrooms play a strategic role in sustainable food systems. They contribute nutritional, environmental, and socioeconomic benefits while supporting circular bioeconomy principles. A sustainable fungal biotechnology model demonstrates that local non-food biomass can first serve as substrates for mushroom cultivation and, subsequently, be recycled as spent mushroom substrates (SMS) for biofuel production, including biogas and ethanol. Selective lignin degradation by fungi facilitates efficient cellulose conversion, enhancing production efficiency. Mushroom cultivation requires relatively low land, water, and energy use while reducing greenhouse gas emissions and promoting the reutilization of agricultural waste. By integrating molecular insights, optimized cultivation practices, strict hygiene protocols, and advanced monitoring systems, the mushroom industry can achieve comprehensive microbial risk management. Therefore, continued interdisciplinary efforts and technological advancements to control microbial contamination are vital for extending shelf life, reducing food waste, and enhancing access to nutrient-rich foods. These efforts contribute to more sustainable food systems and global food security, ensuring the long-term sustainability and resilience of the worldwide mushroom industry. Promoting sustainable mushroom production methods will boost food safety while supporting rural livelihoods and circular bioeconomy initiatives.
